# Risk factors for obstetric anal sphincter injuries at vaginal birth after caesarean: a retrospective cohort study

**DOI:** 10.1007/s00192-019-03978-x

**Published:** 2019-07-02

**Authors:** Joanna C. D’Souza, Ash Monga, Douglas G. Tincello

**Affiliations:** 1 Faculty of Medicine, University of Southampton, University Hospitals Southampton NHS Foundation Trust, Tremona Road, Southampton, SO16 6YD UK; 2 Department of Urogynaecology, University Hospitals Southampton NHS Foundation Trust, Princess Anne Hospital, Coxford Road, Southampton, SO16 5YA UK; 3 Department of Health Sciences, College of Life Sciences, University of Leicester, University Road, Leicester, LE1 7RH UK

**Keywords:** Vaginal birth after caesarean section (VBAC), Obstetric anal sphincter injuries (OASIS), Perineal trauma, Mediolateral episiotomy (MLE)

## Abstract

**Introduction and hypothesis:**

Vaginal birth after caesarean (VBAC) is associated with an increased risk of obstetric anal sphincter injuries (OASIS). However, specific factors that influence the risk of OASIS at VBAC have not been studied, particularly whether there are specific baseline characteristics of the first delivery which affect the subsequent perineal outcomes.

**Methods:**

Retrospective analysis of prospectively collected data from University of Southampton NHS Foundation Trusts’ maternity database. This included secundiparous women with a previous caesarean delivery (CS) who achieved a singleton, term, cephalic vaginal delivery from 2004 to 2014. Univariate analysis compared maternal, intrapartum and neonatal factors of those who suffered OASIS at VBAC with those who did not. A binary logistic regression model calculated the adjusted, independent odds ratio (OR) of OASIS.

**Results:**

A total of 1375 women met the inclusion criteria. The OASIS rate was 8.1%, a 1.4-fold increase compared with primiparous women [difference 2.4% (95% CI 1.1, 3.6)]. Those sustaining OASIS at VBAC were older (*p* = 0.011) and had infants of greater birth weight at initial caesarean (*p* < 0.001) and VBAC (*p* = 0.04). Analysis of odds ratios revealed that mediolateral episiotomy (MLE) at VBAC halved the risk of OASIS [37.5% VBAC with OASIS vs. 52.2% VBAC without OASIS (OR 0.51, 95% CI 0.32–0.81)], whereas an urgent CS at initial delivery doubled the risk [52.3% VBAC with OASIS vs. 34.9% VBAC without OASIS (OR 2.05, 95% CI 1.31–3.21)].

**Conclusions:**

Advanced maternal age, increased infant birth weight and an urgent category of initial CS increase the risk of OASIS at VBAC, whereas MLE is protective.

## Introduction

More than 85% of women sustain some form of perineal trauma during vaginal childbirth in the UK [1]. Obstetric anal sphincter injuries (OASIS), i.e. third- and fourth-degree perineal tears, involve the anal sphincter resulting in significant morbidity in women and are a contributory factor to longer-term anal incontinence and faecal urgency [2]. The rates of OASIS have increased in the UK from 1.8% in 2000 to 5.9% in 2011, and a recent multicentre survey revealed a median national OASIS rate of 2.85% (0–8%) [3, 4]. Risk factors associated with the development of OASIS are nulliparity, infant macrosomia (> 4 kg), induction of labour, prolonged second stage of labour, maternal age and operative vaginal delivery (OVD) [5].

There is a consensus that women who delivered previously with single, uncomplicated caesarean section (CS), and with otherwise uncomplicated current pregnancy, should be encouraged to attempt a vaginal delivery [6, 7].

Although success rates of planned vaginal birth after caesarean section (VBAC) have been quoted to be 63.4–75%, there has been a reported overall decline in attempts to labour after a previous CS [7–9]. This, accompanied by rising rates of primary caesarean, has been a significant driver for the increased CS rate [9].

Research has shown an association between VBAC and an increased risk of OASIS compared with both primparous (adjusted OR 1.42, 95% CI 1.25–1.6. *p* < 0.001) and multiparous (OR 13.6; 95% CI, 4.7–39.3; *p* < 0.001) women [10, 11]. VBAC delivery is also associated with an increased rate of OVD compared with primiparous delivery (39% vs. 30%, OR 1.15, 95% CI 1.01–1.3, *p* < 0.0001) [9]. This may in part be due to the accoucher’s awareness of the risks that prolongation of the second stage of labour have on the risk of uterine scar rupture. Furthermore, OVD further potentiates the risk of OASIS at VBAC, especially with the use of forceps (58% OASIS with forceps at VBAC versus 33% vacuum, *p* = 0.001) [9]. It was also speculated that the increased rate of complicated delivery and OASIS was due to the relative cephalopelvic disproportion as the indication for the initial CS [12]. It has been suggested that risk factors which led to the initial caesarean are carried over to subsequent delivery [13]. Additionally, these are possibly intensified because of more propulsive, secundiparous contractions coupled with a ‘nulliparous’ perineum [12, 14].

Previous studies relied on administrative coding data, which are known to be prone to error, rather than extracting information directly from individual case records [3]. Additionally, previous work has not studied the influence of baseline characteristics or first labour events on the risk of OASIS at subsequent delivery [9]. The objective of this research is therefore to evaluate whether specific baseline characteristics and urgency of caesarean at first delivery affect subsequent vaginal birthing outcomes, especially regarding sustaining OASIS.

## Methods

This is a retrospective cohort study carried out through retrospective analysis of prospectively collected data from the University of Southampton NHS Foundation Trusts’ maternity database. Only anonymised data were used, so informed consent was not required. Data included all secundiparous women with previous CS who achieved a singleton, term, cephalic vaginal delivery at the Trust from January 2004 to December 2014.

Data were extracted electronically from a maternity database to include all women within the study period having delivered vaginally after one previous CS. Women who were delivered by repeat CS, breech delivery or who delivered pre-term were excluded from analysis. Data were then extracted from each of the individual electronic maternity records of those meeting the inclusion criteria. This method of data extraction adjusted for any potential data collection inaccuracies.

We calculated the rate of OASIS and all other forms of perineal trauma among the included women. The degree of OASIS was categorised using the Royal College of Obstetricians and Gynaecologists (RCOG) classification (see Table 1) [15]. Category of CS was classified using the National Institute for Health and Care Excellence (NICE) ‘Caesarean section’ clinical guideline number 132 (see Table 2) and in this study an ‘urgent’ CS was defined as a category 1 or 2 CS [16, 17]. Maternal and neonatal factors were compared between those women who sustained OASIS and those who did not in univariate analysis. Factors reaching statistical significance in this analysis (*p* < 0.05), and those of borderline significance (*p* < 0.2) where previous literature suggested a relationship, were entered into binary logistic regression to calculate the adjusted, independent odds ratio (OR) of OASIS.

**Table 1:** Classification and incidence of obstetric anal sphincter injury (OASI)

Type of OASIS	Count	Percentage of all OASIS
**3a** – < 50% of EAS involved **3b** – ≥ 50% of EAS involved **3c** – EAS and IAS involvement **4th**– 3c + rectal mucosa	46 51 11 4	41.1% 45.5% 9.8% 3.6%
*Total*	**112**	

EAS = external anal sphincter, IAS = internal anal sphincter

**Table 2:** Category of caesarean section

Urgency	Category	Definition
Maternal or fetal compromise	1 2	immediate threat to the life of the woman or fetus maternal or fetal compromise which is not immediately life-threatening
No maternal or fetal compromise	3 4	no maternal or fetal compromise but needs early delivery delivery timed to suit woman or staff

The data were analysed using IBM SPSS v.22 (IBM Corp., 2016). The Kolmogorov-Smirnov test was used to determine the distribution of continuous data. Data are presented as mean (standard deviation) or median (range) as appropriate for normal and non-normal distributions, or as number (%). Normally distributed data were compared using independent samples t-test and non-normally distributed data by the Mann-Whitney U test. Categorical data were analysed using the chi-square test. For comparisons between continuous and categorical data, parametrically distributed data were analysed using the one-way ANOVA and non-parametric data by the Kruskall-Wallis test. Statistical significance was defined as *p* < 0.05. In all analyses, data on third- and fourth-degree OASIS were combined.

Permission to undertake the research was granted by our sponsor, University Hospital Southampton NHS Foundation Trust, under registration no. RHM O&G0234. The study was granted full ethical approval by the Health Research Authority North West-Preston Research Ethics Committee; reference no. 15/NW/0782. Patient consent was not required as this was a retrospective data analysis with no direct patient contact.

## Results

**Fig. 1:**
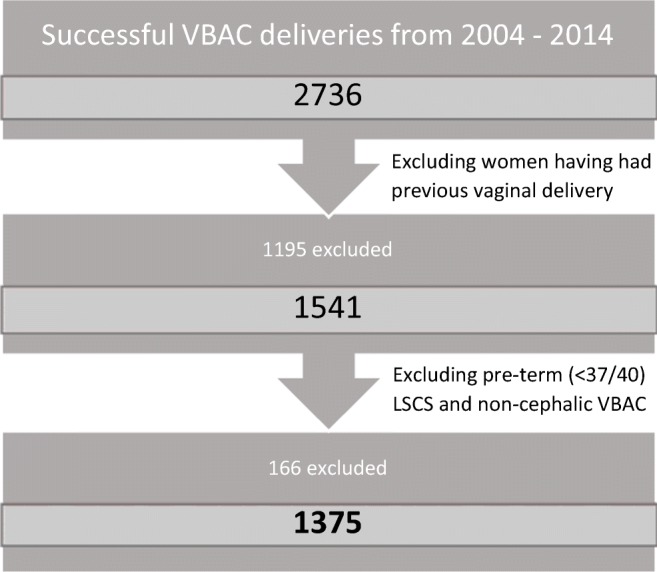
Schematic diagram representing patient numbers

In the 11-year time period there were 2736 successful, singleton VBAC deliveries. The approximated VBAC success rate for the study period was 70.0%. After excluding all those who did not fit the inclusion criteria (see Fig. 1), the study population was 1375. The prevalence of OASIS was 8.1% (112/1375), of which the vast majority had either 3a or 3b tears (41.1% and 45.5%, respectively) (see Table 1).

The hospital’s contemporaneous birthing outcomes revealed an overall OASIS rate of 3.1%, including a multiparous OASIS rate of 1.2% and a primiparous OASIS rate of 5.8%. Compared with the primiparous OASIS rate, secundiparous women at VBAC were at a 1.4-fold greater risk [8.1% vs. 5.8%, difference 2.4% (difference 2.4%, 95% CI 1.1, 3.6)]. Secundiparous women were at significantly greater risk than other multiparous women with prior vaginal deliveries (difference 7.0, 95% CI 6.4, 7.6), representing a 6.8-fold increase.

Of those not sustaining an OASI, 816 (64.6%) had no spontaneous trauma. However, 77.5% had a mediolateral episiotomy (MLE). The overall MLE rate was 50.8%, which is 2.3 times greater than the Trust’s rate for the concurrent period (50.8% vs. 22.3%). The rate of MLE at normal vaginal delivery (NVD) was 20.2%, whereas 88.2% at OVD (94.5% with forceps, 75.0% with ventouse). The next common birthing outcome was a second-degree tear (see Table 3).

**Table 3:** Perineal outcomes of those not sustaining OASIS

Perineal Condition	Count	Percentage of all births (*n* = 1375)	Percentage that had episiotomy
No spontaneous trauma	816 (64.6%)	59.3%	632 (77.5%)
1st	54 (4.3%)	3.9%	3 (5.6%)
2nd	393 (31.1%)	28.6%	23 (5.9%)
Total	1263		

Univariate analyses are shown in Tables 4 to 6, to identify factors associated with OASIS in this cohort. Women sustaining OASIS at VBAC were significantly older than those who did not; 68.7% of the women sustaining OASIS at VBAC were over the age of 30 years vs. 57.5% of the non-OASIS population, although this distribution difference was not significant. We identified large differences in the frequency of OASIS among women of non-Caucasian ethnicity (1.5-fold more Asian and 2.7-fold fewer Black women) although these did not reach statistical significance.

**Table 4:** Maternal demographics

		VBAC with OASIS	VBAC, no OASIS	*p* value
Age		(*n* = 112)	(*n* = 1263)	
	Median	32.3 (21.0–43.6)	31.0 (17.3–45.9)	***p*** **= 0.011**^**a**^
	By age category:			
	<20 20–25 25–30 30–35 35–40 >40	0 (0.0%) 9 (8.0%) 26 (23.2%) 52 (46.4%) 22 (19.6%) 3 (2.7%)	13 (1.0%) 199 (15.8%) 324 (25.7%) 456 (36.1%) 232 (18.4%) 38 (3.0%)	*p* = 0.002^**b**^
Ethnicity		(*n* = 111)	(*n* = 1231)	
	Caucasian Asian Black	95 (85.5%) 15 (13.5%) 1 (0.9%)	1090 (88.5%) 111 (9.0%) 30 (2.4%)	***p*** **= 0.189**^**c**^

^a^Mann-Whitney U test (non-parametric data), ^b^ one-way ANOVA, ^c^ chi-square

**p ≤ 0.05** (p values in bold type)—used in the binary logistic regression

**Table 5:** Information regarding the VBAC delivery

		VBAC with OASIS (*n* = 112)	VBAC, no OASIS (*n* = 1263)	*p* value
Birth weight (g)	Mean	3642.2 (±488.26)	3465.6 (±470.27)	*p* < 0.001^a^
	% over 4Kg	27 (24.1%)	166 (13.1%)	***p*** = **0.001**^b^
Operative vaginal delivery (OVD)	As a percentage of all deliveries	51 (45.5%)	568 (45.0%)	*p* = 0.918^b^
	Comparison of instrument			
	- Forceps	41 (80.4%)	378 (66.5%)	*p* = 0.043^b^
	- Ventouse	10 (19.6%)	190 (33.5%)	
Episiotomy	Overall rate	42 (37.5%)	657 (52.2%)	***p*** = **0.003**^b^
	OVD episiotomy rate	*n* = 51	n = 568	
		36 (70.6%)	510 (89.8%)	*p* < 0.001^b^
	- Forceps	34 (82.9%)	362 (95.8%)	*p* = 0.001^b^
	- Ventouse	2 (20.0%)	148 (77.9%)	*p* < 0.001^b^
	NVD episiotomy rate	*n* = 61	n = 695	
		6 (9.8%)	147 (21.2%)	*p* = 0.035^b^
Gestation Post-term (>40 weeks)		69 (61.6%)	717 (56.8%)	*p* = 0.326^b^
Induction of labour		20 (17.9%)	216 (17.1%)	*p* = 0.839^b^
Use of regional anaesthesia		37 (33.0%)	506 (36.8%)	***p*** **= 0.145** ^b^
Head position (if OP)		4 (3.6%)	38 (3.0%)	*p* = 0.744^b^
Shoulder Dystocia		5 (4.5%)	23 (1.8%)	***p*** **= 0.058** ^b^
Length of 2nd stage (mins)	Median			
	- Active	45 (4–148)	49 (1–211)	*p* = 0.845^c^
	- Total	50 (6–213)	60 (1–554)	*p* = 0.995^c^

^a^Independent t-test (parametric), ^b^chi-square, ^c^Mann-Whitney U test (non-parametric data)

***p*** **≤ 0.05** (*p* values in bold type)—used in the binary logistic regression

**Table 6:** Information regarding the initial caesarean delivery

		VBAC with OASIS (*n* = 112)	VBAC, no OASIS (*n* = 1263)	*p* value
Gestation Post-term (>40 weeks)		52 (46.4%)	624 (49.4%)	*p* = 0.546^**a**^
Birth weight at LSCS (g)	Mean	3557 (±543.53)	3450 (±527.50)	***p*** **= 0.04**^**b**^
	% over 4Kg	22 (19.6%)	178 (14.2%)	*p* = 0.120^**a**^
LSCS in labour		91 (81.3%)	980 (77.6%)	*p* = 0.371^**a**^
Induction of labour		31 (27.7%)	344 (27.2%)	*p* = 0.920^**a**^
Cervical dilatation (cm)		(*n* = 82)	(*n* = 936)	
(at time of CS decision)	Median	6 (0–10)	5 (0–10)	p = 0.336^c^
	10 cm dilated	8 (9.8%)	108 (11.5%)	p = 0.624^a^
Non-cephalic presentation		22 (19.6%)	325 (25.7%)	*p* = 0.155^**a**^
Category of LSCS^	Overall comparison			*p* = 0.007^**a**^
	Category 1 Category 2 Category 3 Category 4	7 (8.0%) 39 (44.3%) 29 (33.0%) 13 (14.7%)	74 (7.8%) 259 (27.1%) 419 (43.9%) 202 (21.2%)	
	Urgent CS (1 + 2)	46 (52.3%)	333 (34.9%)	***p*** **= 0.001**^**a**^

^a^Chi-square, ^b^ independent t-test (parametric), ^c^ Mann-Whitney U test (non-parametric data)

***p*** **≤ 0.05** (*p* values in bold type)—used in the binary logistic regression

^Initial caesarean data not available for all births; for 78.6% (88/112) OASIS at VBAC, 75.5% (954/1263) no OASIS

Those with OASIS had significantly heavier babies, with a significantly greater proportion weighing > 4 kg.

Operative vaginal delivery (OVD) at VBAC was 2.4-fold more likely than the Trust’s approximated OVD rate (45.0% vs. 18.9%). While there was no difference in whether the VBAC deliveries were operative, the women sustaining OASIS at VBAC had significantly more forceps deliveries.

Women without OASIS were significantly more likely to have had an MLE. This difference was seen regardless of the type of delivery.

There were no significant differences when analysing the foetal head position [whether occiput posterior (OP) or not], length of second stage of labour, use of regional anaesthesia during second stage or whether the VBAC was post-term or induced. Although the difference was not significant, those sustaining OASIS were 2.5-fold more likely to have delivered a baby with shoulder dystocia than those without OASIS.

When comparing those that sustained OASIS at VBAC with those that did not, there was no difference in whether the initial CS was post-term or whether the CS followed an induction of labour, or in overall cervical dilation at the time of CS decision or whether fully dilated at CS decision (see Table 6).

Of all VBAC deliveries, 78.0% (1071/1375) had an initial CS in labour. Those sustaining OASIS at VBAC were more likely to have been in labour at the initial CS. The OASIS rate of those that had an initial CS whilst in labour was 8.5% (91/1071) compared with 6.9% (21/304) of those that were not in labour at initial CS (*p* = 0.371).

Although there was no difference if at initial CS there was a non-cephalic presentation, those presenting this way were 1.3-fold less likely to have OASIS at VBAC.

Those that had OASIS at VBAC had significantly heavier babies at the initial CS, but no difference was seen in the proportion of those that had a birth weight > 4 kg.

There was a significant difference when comparing the overall categories of CS; moreover, those sustaining OASIS at VBAC were 1.5-fold more likely to have an urgent CS.

The factors which remained independently associated with the risk of OASIS after binary logistic regression are shown in Table 7. These included the age of the mother, birth weight at VBAC, whether a MLE was performed and whether the initial CS was urgent (category 1 or 2; see Table 2). The same factors were seen when including all statistically significant outcomes (i.e. *p* ≤ 0.05) vs. those that had a significance of *p* < 0.2. Regression analysis including birth weight as a continuous variable gave an OR increase of 1.001 (95% CI, 1.000–1.001. *p* = 0.001) per gram of increased birth weight. Results presented in the table show birth weight dichotomised into ‘> 4 kg or not’ to make this easier to interpret. The analysis of odds ratios revealed that MLE at VBAC more than halved the risk of OASIS, whereas an urgent CS at initial delivery more than doubled the risk (see Table 7).

**Table 7:** Factors independently associated with the risk of OASIS at VBAC after binary logistic regression

	VBAC with OASIS (*n* = 112)	VBAC, no OASIS (*n* = 1263)	OR	95% CI	p value
Maternal age (years)	32.3 (21.0–43.6)	31.0 (17.3–45.9)	1.054	1.008–1.102	0.020
If baby >4Kg (%)	27 (24.1%)	166 (13.1%)	2.146	1.091–3.426	0.006
Episiotomy (%)	42 (37.5%)	657 (52.2%)	0.511	0.321–0.813	0.005
Emergency CS (%)	46* (52.3%)	333* (34.9%)	2.054	1.313–3.213	0.002

*Initial caesarean data not available for all births; for 78.6% (88/112) OASIS at VBAC, 75.5% (954/1263) no OASIS at VBAC

## Discussion

### Main findings

This study, carried out between January 2004 and December 2014 within the University of Southampton NHS Foundation Trust, aimed to assess whether women having VBAC are at increased risk of sustaining OASIS and whether specific baseline characteristics and indication for initial CS affect subsequent birthing outcomes.

The main finding was that a VBAC delivery does significantly increase the likelihood of sustaining OASIS; this more so than at primiparous vaginal birth, which is in line with previous findings [10, 12, 13]. As with other OASIS studies, this study found an association of an increased risk of OASIS with foetal macrosomia and increased maternal age [5]. Additionally, MLE was found to be strongly protective against OASIS at VBAC. Furthermore, this research has revealed that a previous urgent caesarean is associated with significantly increased risk of OASIS at VBAC.

### Strengths and limitations

This study’s strength lies in the fact that the available information was collected manually by looking at the electronic documentation of every woman undergoing secundiparous VBAC during the study period. This removed any potential inaccuracies associated with incomplete or incorrect coding of electronically devised data sets found which other studies have encountered [4, 8]. However, the information concerning the initial CS was missing in some cases because of the birth taking place prior to the electronic documentation or at a different Trust. The authors decided not to include the Category 3 CS as ‘Emergency’ as the majority (85.3%) of documented cases were due to failure to progress of the first stage, non-cephalic presentation or maternal infirmity, i.e. reasons not related to the pelvic outlet or pressure on the perineum. It would have been interesting to analyse the CS category decision making in more detail, but due to incomplete and unreliable documentation this was not possible. The VBAC rate (as a percentage of total VBAC deliveries including repeat caesareans which were unplanned) was a predicted value, and as such was unable to establish the VBAC failure rate. It would be useful to have this information to analyse the reason for repeat CS and to see whether this correlates with why an initial CS was required.

### Interpretation

Although it could be reasonable to assume the risk of OASIS to a woman undergoing VBAC delivery is similar to that of a nulliparous patient, this study found not only the OASIS rate but also the rate of OVD to be increased. This supports earlier studies as well as speculation about a relative cephalopelvic disproportion and risk of such carried over from previous delivery. Like Hehir et al., this study found forceps delivery to cause a greater increase in risk of OASIS than vacuum extraction, especially when no MLE is performed [10, 11, 20]. This is not surprising because of the additional force exerted on the perineum to aid the delivery of the foetal head.

Previous studies have shown a negative correlation between perineal length and risk of OASIS. Additionally, Asian women have been found to be at increased risk of severe perineal trauma but the causation has been disputed: whether this is due to anatomical differences in perineal length or other factors such as differences in pelvic shape or tissue composition [14, 18, 19]. An earlier study found an element of protection against sphincter tears in African-American women, but not of statistical significance [13]. Although documentation of anatomical variations was not within the remit of our study, the findings supported previous research as Asian women were at increased risk, whereas Black women were at decreased risk.

The overall MLE rate in our cohort was far greater, and more so in the cohort not sustaining OASIS, than the national rate of 20.2% quoted for all deliveries by the respondents in a national survey of maternity units (response rate 82%). However, it seems unlikely the non-response bias would greatly change this figure [4]. This therefore is suggestive of MLE being protective against OASIS at VBAC. A systematic review revealed a 40–50% risk reduction of OASIS compared with spontaneous tears through relieving the pressure on the central posterior perineum via MLE [20]. Our regression model showed the same outcome. The study also found significantly fewer patients sustained OASIS if an MLE was performed, regardless of whether the delivery was an OVD or not. However, 6.8 episiotomies would need to be performed to prevent one OASIS [NNT = 1/ARR = 1/ (0.375–0.522) = −6.8].

A validated prediction model for successful VBAC highlights the indication of initial CS (arrested dilatation or failure of descent) as a negative predictor of success at VBAC [21]. Although widely used, this measure of “success” is focussed solely on achieving vaginal delivery, with no interest in the impact of the delivery on the perineum. Thus our study provides additional valuable information. It is the first VBAC study which examined perineal trauma outcomes at subsequent birth and examined factors associated with the initial caesarean birth which may influence perineal outcome, particularly OASIS. We were able to establish the association between urgency of initial delivery and increased likelihood of severe perineal trauma at subsequent delivery.

## Conclusion

This study has shown that secudiparous VBAC delivery is associated with a significantly increased risk of OASIS, especially if the initial CS was urgent. Additional risk factors were increased maternal age and increasing birthweight. The current patient pathway for VBAC delivery makes no reference to these risks, so our findings provide new data to improve counselling of potential VBAC patients, provision of information and candidate selection [7]. Currently, the only basis of whether a VBAC delivery is “successful” is if the infant is born vaginally. More consideration needs to be made about the potential impact of VBAC delivery on the perineum and the resultant effects this may have on long-term physical, social and psychological wellbeing of patients.
